# Radiomics machine learning model based on multi-sequence MRI for predicting Ki-67 expression level in glioblastoma

**DOI:** 10.3389/fmolb.2026.1876065

**Published:** 2026-09-14

**Authors:** Juan Ma, Palidanmu Wumaier, Gulijianaiti Maiamaituxun, Maiwulanjiang Amuti, Mireguli Damaola, Abudoumijiti Aji, Lijuan Da, Xin Qiao, Mayidili Nijiati, Diliaremu Aihaiti, Chunhui Jiang

**Affiliations:** 1 Department of Radiology, The Fourth Affiliated Hospital of Xinjiang Medical University, Urumqi, China; 2 Department of Radiology, The First People’s Hospital of Kashi Prefecture, Kashi, China; 3 Department of Neurology, The First People’s Hospital of Kashi Prefecture, Kashi, China; 4 Department of Neurosurgery, The First People’s Hospital of Kashi Prefecture, Kashi, China; 5 Department of Research Collaboration, R&D Center, Beijing Deepwise & League of PHD Technology Co., Ltd, Beijing, China; 6 First Affiliated Hospital of Xinjiang Medical University Department of Imaging Center, Urumqi, China

**Keywords:** glioblastoma, Ki-67 proliferation index, machine learning, multi-sequence MRI, radiomics

## Abstract

**Introduction:**

Glioblastoma (GBM) is the most aggressive primary malignant brain tumor in adults, with median overall survival around 15 months. The Ki- 67 proliferation index is an important marker of proliferative activity and has prognostic relevance in GBM; however, its assessment requires surgical tissue and may be affected by sampling bias. Developing a non-invasive preoperative method to estimate Ki-67 expression may provide adjunctive information for risk assessment, while it should not be regarded as a stand-alone determinant of treatment decisions.

**Methods:**

This retrospective study included 269 histologically confirmed GBM patients from The Fourth Affiliated Hospital of Xinjiang Medical University. Patients were classified into low (n = 61) and high (n = 208) Ki-67 expression groups using a 20% threshold, selected according to published GBM Ki-67 stratification literature and local pathology reporting practice. Multi-sequence MRI scans were acquired at 3.0T, including T1-weighted imaging (T1WI), T2-weighted imaging (T2WI), fluid-attenuated inversion recovery (FLAIR), and contrast-enhanced T1WI (CET1WI). A total of 8,612 radiomic features were extracted from three-dimensional tumor volumes across all sequences. After reducing dimensionality via Pearson correlation coefficient filtering and LASSO regression, machine learning models were built using BernoulliNB, GaussianNB, SVM, and AdaBoost classifiers for each sequence independently and a Combined model integrating all four sequences. Model performance metrics included AUC, accuracy, sensitivity, specificity, F1 score, calibration curves, and decision curve analysis (DCA). SHapley Additive exPlanations (SHAP) were used to interpret model predictions.

**Results:**

Baseline demographics (gender, BMI, age) did not differ significantly between groups. The Combined model integrating all sequences achieved the highest predictive accuracy, with AUCs of 0.915 (95% CI: 0.874–0.956) in the training set and 0.746 (95% CI: 0.614–0.879) in the test set, outperforming single-sequence models. Notably, the Combined model attained sensitivity of 0.930 and negative predictive value of 0.972 in training, indicating strong identification of high Ki-67 expression. Calibration curves and DCA demonstrated good model calibration and clinical utility.

**Discussion:**

A radiomics-based multi-sequence MRI machine learning model may serve as a non-invasive adjunct for preoperative Ki-67 estimation in GBM. The combined sequence approach outperformed single modalities, and SHAP analysis enhanced interpretability; however, clinical application requires external validation and outcome-based evidence before use in treatment decision-making.

## 1 Introduction

Glioblastoma (GBM) is the most prevalent and aggressive primary malignant brain tumor in adults, characterized by relentless progression and a dismal prognosis despite multimodal treatment comprising maximal safe surgical resection, radiotherapy, and temozolomide chemotherapy (Campos et al., 2016). The median overall survival for patients with GBM remains approximately 15 months, a statistic that has seen only marginal improvement over the past 2 decades (Vychopen et al., 2024). This poor outcome is largely attributable to the profound intratumoral heterogeneity of GBM, which manifests at both the genetic and cellular levels, driving therapeutic resistance and inevitable recurrence (Patel et al., 2014). Consequently, there is an urgent need for robust biomarkers that can stratify patients according to tumor biology and guide personalized therapeutic strategies.

The Ki-67 proliferation index is a well-established immunohistochemical marker that reflects the fraction of actively proliferating tumor cells and serves as a prognostic indicator in GBM4 (Bai et al., 2023). A high Ki-67 expression level has been associated with more aggressive tumor behavior, recurrence risk, and shorter overall survival (Jin et al., 2011). However, Ki-67 is not an established stand-alone biomarker for selecting GBM treatment, and the current assessment relies on surgical biopsy or resection, which is subject to sampling bias due to the heterogeneous nature of GBM6 (Dahlrot et al., 2021; Zhu et al., 2024). Therefore, a non-invasive method for preoperatively estimating Ki-67 expression may provide useful adjunctive information for biological risk assessment and patient stratification, but should be interpreted together with histopathological, molecular, and clinical findings.

Radiomics, a high-throughput computational approach that extracts a large number of quantitative imaging features from medical images, has emerged as a powerful tool for capturing tumor phenotypic characteristics that are imperceptible to the human eye (Gillies et al., 2016). These features, which describe tumor texture, shape, and intensity, can be integrated with machine learning algorithms to construct predictive models. Multi-parametric magnetic resonance imaging (MRI), encompassing sequences such as T1-weighted imaging (T1WI), T2-weighted imaging (T2WI), fluid-attenuated inversion recovery (FLAIR), and contrast-enhanced T1WI (CET1WI), provides a comprehensive view of tumor morphology, edema, necrosis, and vascularity (Gong et al., 2024). The combination of radiomics from multi-sequence MRI offers a more holistic characterization of the tumor microenvironment, potentially improving predictive accuracy compared to single-sequence approaches (Mang et al., 2020).

Despite the promise of radiomics, previous studies on predicting Ki-67 in GBM have often been limited by small sample sizes, reliance on single or dual MRI sequences, and a lack of rigorous model validation. Furthermore, the interpretability of these complex models, which is crucial for clinical translation, has been largely unexplored. To address these gaps, this study aims to develop and internally validate a radiomics machine learning model based on multi-sequence MRI (T1WI, T2WI, FLAIR, and CET1WI) for the preoperative prediction of Ki-67 expression levels in GBM. A key methodological feature of this work is the systematic integration of features from all four sequences to construct a combined model, alongside the application of SHapley Additive exPlanations (SHAP) analysis to elucidate the imaging basis of the model’s predictions. The primary objective is to evaluate whether multi-sequence MRI radiomics can provide an interpretable, non-invasive adjunct for Ki-67 stratification rather than to replace pathological assessment or directly guide treatment decisions.

## 2 Methods

The study, conducted at The Fourth Affiliated Hospital of Xinjiang Medical University, received ethical approval from their respective institutional review boards. As it was a retrospective study, written informed consent was waived.

### 2.1 Patient population

A total of 269 patients were included in this retrospective study.

Inclusion criteria were as follows: (1) histologically verified GBM; (2) Ki-67 evaluated by surgical histopathology; (3) age 18 years or older; (4) availability of axial T1-weighted imaging (T1WI), T2-weighted imaging (T2WI), fluid-attenuated inversion recovery (FLAIR), and contrast-enhanced T1-weighted imaging (CE-T1WI); (5) absence of prior brain tumors; and (6) availability of relevant clinical information.

Exclusion criteria (1) lack of surgical pathology; (2) unavailability of axial T1WI, T2WI, FLAIR, CE-T1WI; (3) poor image quality; (4) previous treatment history.

Ki-67 pathological assessment was based on immunohistochemical evaluation of surgical specimens in routine clinical pathology reports. The Ki-67 labeling index was recorded as the percentage of positively stained tumor nuclei in representative diagnostic tumor tissue. Because archival information regarding whole-slide digital quantification, exact hotspot counting protocol, and the number of tissue blocks was not consistently available for all patients, Ki-67 was analyzed as a patient-level pathological label. A threshold of 20% was used to dichotomize Ki-67 expression into low (≤20%) and high (>20%) groups, consistent with published GBM Ki-67 stratification literature and local reporting practice, while recognizing that Ki-67 is a continuous biological variable (Bai et al., 2023; Dahlrot et al., 2021; Zhu et al., 2024).

### 2.2 Imaging data acquisition

The study utilized three 3.0-T MRI scanners. Specifically, we utilized the Signa Hdx MR scanner from General Electric (USA). The imaging protocol included axial contrast-enhanced T1-weighted imaging (CE-T1WI), axial T2-weighted imaging (T2WI), axial fluid-attenuated inversion recovery (FLAIR), sagittal T2WI, and contrast-enhanced axial, sagittal, and coronal T1WI sequences. For the axial T1WI sequence, key parameters were set as follows: repetition time (TR) = 200 ms, echo time (TE) = 12 ms, and slice thickness = 6 mm. T2WI image acquisition parameters included TR = 3,900 ms, TE = 120 ms, slice thickness = 6 mm, and a field of view (FOV) of 256 × 256 matrices.

Image retrieval from the picture archiving and communication system (PACS) utilized the Digital Imaging and Communications in Medicine (DICOM) format.

### 2.3 Region of interest (ROI) segmentation

Workflow of the study are presented in Figure 1. Multi-sequence MRI data of glioblastoma patients, including T1-weighted imaging (T1WI), T2-weighted imaging (T2WI), fluid-attenuated inversion recovery (FLAIR), and contrast-enhanced T1-weighted imaging (CET1WI), were imported into the Deepwise Medical Multimodal Research Platform (Version 3.1.3; https://keyan.deepwise.com, Beijing Deepwise BoLian Technology Co., Ltd., Beijing, China). Two experienced radiologists manually delineated the primary tumor lesion slice by slice to generate a three-dimensional volume of interest (VOI). Segmentation strictly followed the principle of complete coverage of lesion boundaries; ambiguous boundary regions were cross-calibrated with multi-sequence images.

**FIGURE 1:**
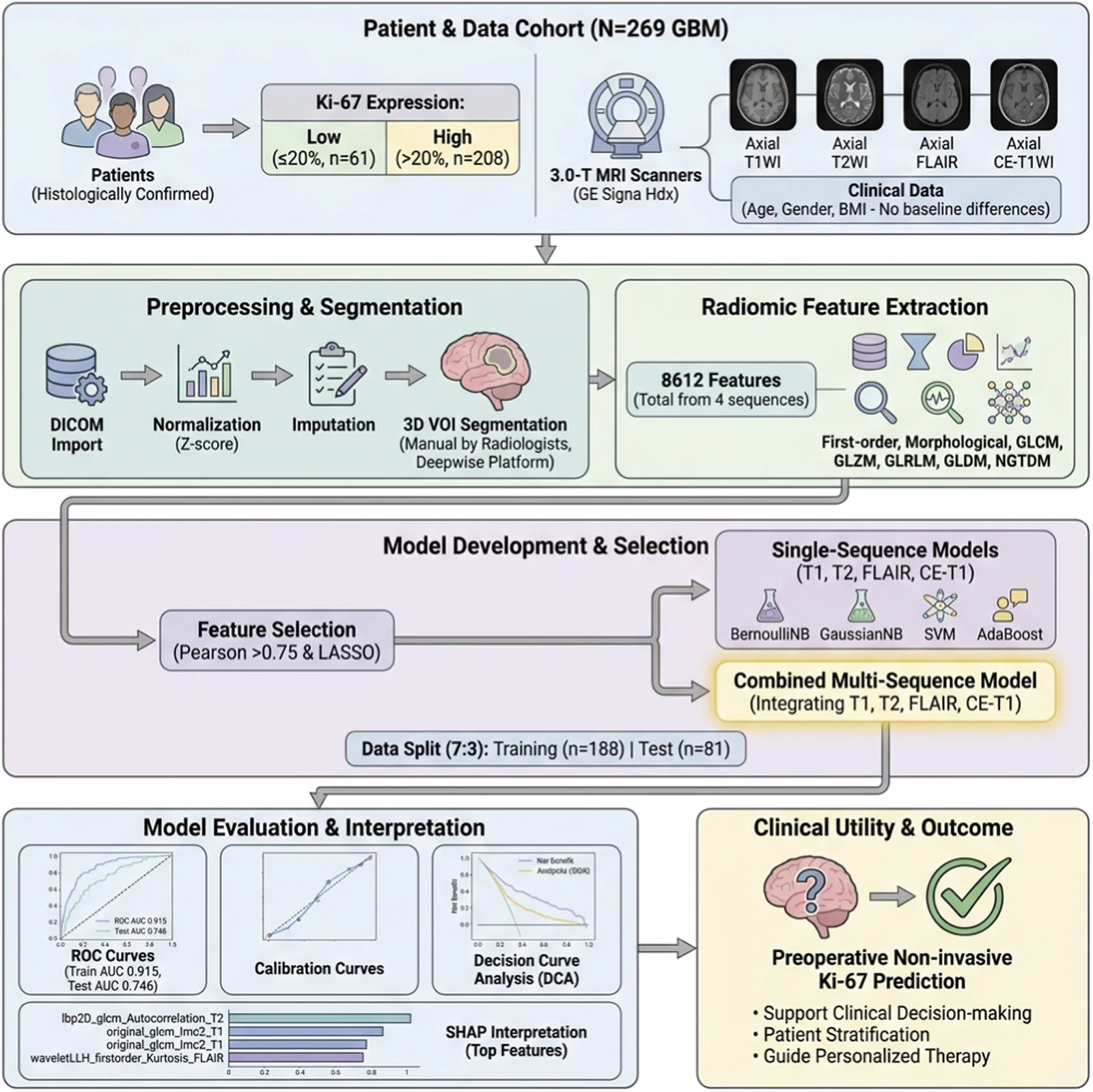
Workflow of the study.

### 2.4 Feature extraction and data preprocessing

Radiomic features were extracted from the VOI using the Deepwise Medical Multimodal Research Platform (Version 3.1.3). Seven categories of features were extracted: first-order statistical features, morphological features, gray-level co-occurrence matrix (GLCM), gray-level zone matrix (GLZM), gray-level run-length matrix (GLRLM), gray-level dependence matrix (GLDM), and neighborhood gray-tone difference matrix (NGTDM). A total of 2,153 features were extracted per sequence, yielding 8,612 radiomic features across four sequences.

A total of 269 study subjects were randomly divided into a training set (n = 188) and a test set (n = 81) at a ratio of 7:3. Missing values in the dataset were imputed using mean substitution. Continuous independent variables were standardized using the Z-score method to achieve a mean of 0 and standard deviation of 1, eliminating dimensional effects and improving model convergence efficiency.

### 2.5 Model construction and evaluation

Highly redundant features were removed using Pearson correlation coefficient with a threshold of 0.75, and key features were selected in combination with LASSO regression. Models were constructed based on BernoulliNB, GaussianNB, SVM, and AdaBoost algorithms for T1, T2, FLAIR, CET1, and the Combined model integrating all four sequences.

Model performance was compared using the area under the curve (AUC), accuracy, precision, recall, F1 score, sensitivity, and specificity. The Combined model was comprehensively evaluated for discrimination, calibration, and clinical net benefit using receiver operating characteristic (ROC) curves, calibration curves, and decision curve analysis (DCA).

## 3 Results

### 3.1 Patient characteristics

The baseline characteristics of all enrolled patients are summarized in Table 1. A total of 269 patients were included in the analysis, stratified into a low Ki-67 expression group (n = 61) and a high Ki-67 expression group (n = 208) based on a threshold of 20%. Regarding gender distribution, the low expression group comprised 34 males (55.7%) and 27 females (44.3%), while the high expression group included 123 males (59.1%) and 85 females (40.9%), with no statistically significant difference observed between the two groups (*P* = 0.745). Body mass index (BMI) was 20.93 ± 2.45 kg/m^2^ in the low expression group and 21.29 ± 2.36 kg/m^2^ in the high expression group, with no significant difference detected (*P* = 0.307). The mean age of patients in the low expression group was 55.85 ± 13.32 years, compared to 54.42 ± 13.40 years in the high expression group, and this difference was not statistically significant (*P* = 0.462). Overall, no significant differences were found in baseline demographic characteristics between the two groups (*P* > 0.05 for all variables), indicating that the two groups were well-matched and comparable at baseline.

**TABLE 1:** Baseline patient characteristics.

Variables	Low expression (<=20%, n = 61) *mean* ± SD, n (%)	High expression (>20%,n = 208) *mean* ± SD, n (%)	*P*
Gender			0.745
Male	34 (55.7)	123 (59.1)	
Female	27 (44.3)	85 (40.9)	
BMI	20.93 ± 2.45	21.29 ± 2.36	0.307
Age (years)	55.85 ± 13.32	54.42 ± 13.40	0.462

### 3.2 Overall model performance

The predictive performance of radiomics models for each sequence in the training and test sets is summarized in Table 2. In the training set, the Combined model achieved the highest AUC of 0.915 (95% CI: 0.874–0.956), outperforming all single-sequence models. In the test set, the Combined model yielded an AUC of 0.746 (95% CI: 0.614–0.879), with overall performance superior to T1, T2, FLAIR, and CET1 single-sequence models. The decrease in AUC from the training to the test set indicates that model robustness should be interpreted cautiously and requires external validation. The Combined model showed a sensitivity of 0.930 and a negative predictive value (NPV) of 0.972 in the training set, indicating high sensitivity for identifying high Ki-67 expression in the derivation cohort. The Brier Score was consistently low across datasets, suggesting favorable accuracy of predicted probabilities.

**TABLE 2:** Performance of radiomics models based on individual sequences and the Combined model for predicting Ki-67 expression in glioblastoma.

Dataset	Sequence	AUC (95% CI)	Accuracy (95% CI)	Precision (95% CI)	Recall (95% CI)	F1 score (95% CI)	Sensitivity (95% CI)	Specificity (95% CI)	PPV (95% CI)	NPV (95% CI)	Brier score
Training (n = 188)	T1	0.771 [0.696–0.845]	0.676 [0.610–0.740]	0.390 [0.320–0.460]	0.744 [0.610–0.870]	0.512 [0.440–0.580]	0.744 [0.610–0.870]	0.655 [0.580–0.730]	0.390 [0.280–0.500]	0.896 [0.840–0.950]	0.235
	T2	0.773 [0.691–0.855]	0.755 [0.690–0.820]	0.467 [0.400–0.540]	0.488 [0.340–0.640]	0.477 [0.410–0.550]	0.488 [0.340–0.640]	0.834 [0.770–0.890]	0.467 [0.320–0.610]	0.846 [0.790–0.910]	0.219
	FLAIR	0.815 [0.742–0.887]	0.654 [0.590–0.720]	0.380 [0.310–0.450]	0.814 [0.760–0.870]	0.519 [0.450–0.590]	0.814 [0.700–0.930]	0.607 [0.530–0.690]	0.380 [0.280–0.480]	0.917 [0.860–0.970]	0.156
	CET1	0.766 [0.694–0.839]	0.766 [0.710–0.830]	0.486 [0.410–0.560]	0.395 [0.330–0.470]	0.436 [0.370–0.510]	0.395 [0.250–0.540]	0.876 [0.820–0.930]	0.486 [0.320–0.650]	0.830 [0.770–0.890]	0.236
	Combined	0.915 [0.874–0.956]	0.771 [0.710–0.830]	0.500 [0.430–0.570]	0.930 [0.890–0.970]	0.650 [0.580–0.720]	0.930 [0.850–1]	0.724 [0.650–0.800]	0.500 [0.390–0.610]	0.972 [0.940–1]	0.161
Validation (n = 81)	T1	0.673 [0.527–0.818]	0.605 [0.500–0.710]	0.306 [0.210–0.410]	0.611 [0.390–0.840]	0.407 [0.300–0.510]	0.611 [0.390–0.840]	0.603 [0.480–0.720]	0.306 [0.160–0.460]	0.844 [0.740–0.950]	0.275
	T2	0.623 [0.470–0.776]	0.654 [0.550–0.760]	0.273 [0.180–0.370]	0.333 [0.120–0.550]	0.300 [0.200–0.400]	0.333 [0.120–0.550]	0.746 [0.640–0.850]	0.273 [0.090–0.460]	0.797 [0.690–0.900]	0.327
	FLAIR	0.713 [0.574–0.853]	0.654 [0.550–0.760]	0.361 [0.260–0.470]	0.722 [0.620–0.820]	0.481 [0.370–0.590]	0.722 [0.520–0.930]	0.635 [0.520–0.750]	0.361 [0.200–0.520]	0.889 [0.800–0.980]	0.159
	CET1	0.499 [0.337–0.662]	0.704 [0.600–0.800]	0.333 [0.230–0.440]	0.333 [0.230–0.440]	0.333 [0.230–0.440]	0.333 [0.120–0.550]	0.810 [0.710–0.910]	0.333 [0.120–0.550]	0.810 [0.710–0.910]	0.348
	Combined	0.746 [0.614–0.879]	0.654 [0.550–0.760]	0.361 [0.260–0.470]	0.722 [0.620–0.820]	0.481 [0.370–0.590]	0.722 [0.520–0.930]	0.635 [0.520–0.750]	0.361 [0.200–0.520]	0.889 [0.800–0.980]	0.179

### 3.3 Evaluation curves of the combined model

Figure 2 presents the evaluation curves of the Combined model. The ROC curves for the training set (Figure 2A) and test set (Figure 2B) visually depict discriminatory ability, although the validation performance was lower than the training performance. The decision curve analysis (Figure 2C) demonstrates that the Combined model provides a greater net clinical benefit than both the “all-intervention” and “no-intervention” strategies across a range of risk thresholds, supporting possible decision-support value rather than direct treatment selection. The calibration curve (Figure 2D) indicates agreement between predicted probabilities and observed outcomes, suggesting acceptable probability estimation by the model.

**FIGURE 2:**
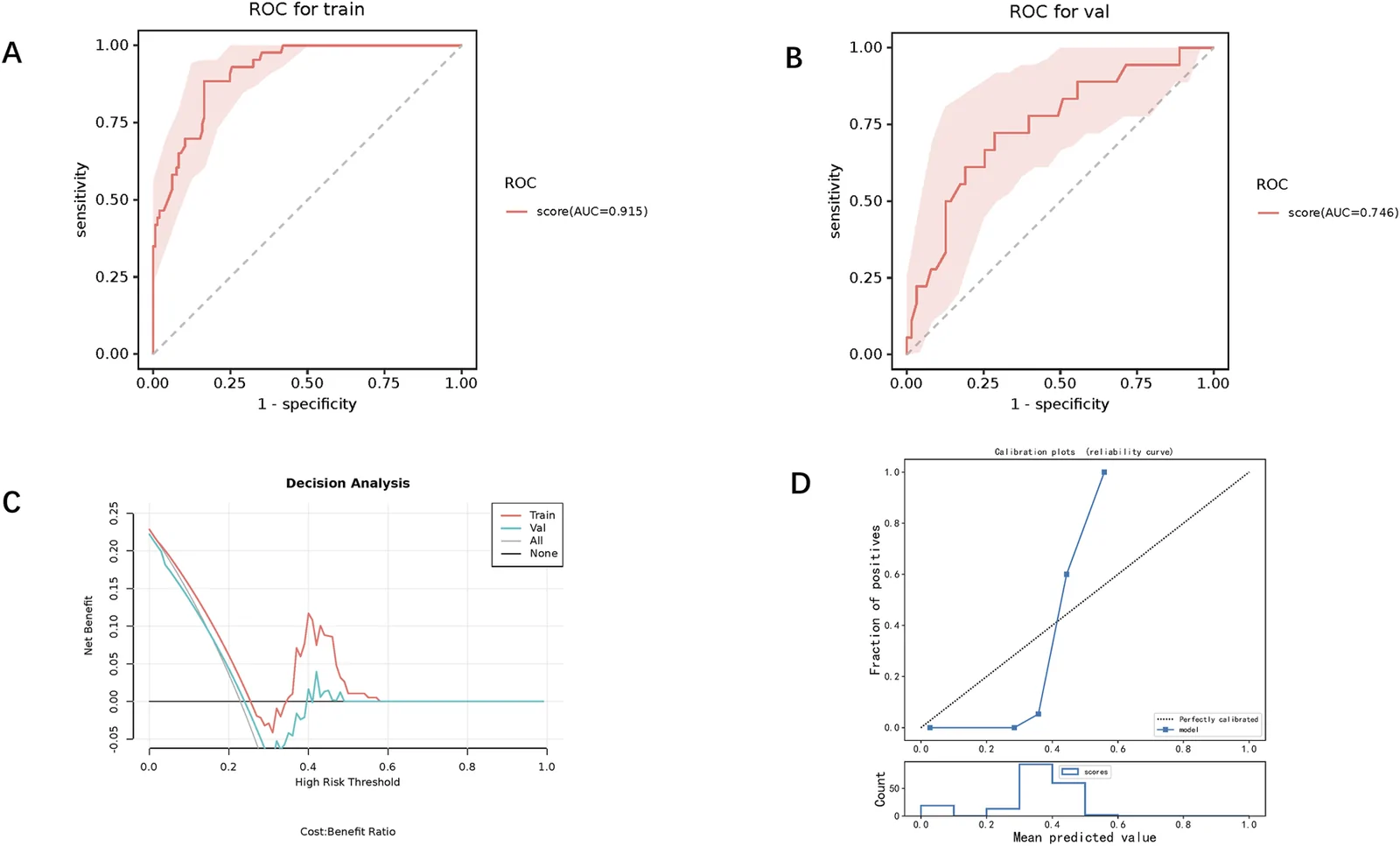
**(A)** ROC for the training cohort; **(B)** ROC for the validation cohort; **(C)** Decision analysis; **(D)** Claibration curve.

### 3.4 Confusion matrices of testing and validation cohorts

To further characterize model performance, confusion matrices summarizing prediction outcomes in both training and validation cohorts are depicted in Figure 3. In the training cohort, the model correctly classified 143 out of 145 patients with high Ki-67 expression (>20%) (true positives), demonstrating a very low false negative rate. Among patients with low Ki-67 expression (≤20%), 18 were correctly identified (true negatives), though 25 were misclassified, indicating room for improvement in identifying low-expression cases. Comparable performance was observed in the validation cohort, Figure 3. These confusion matrices provide intuitive visual confirmation of the model’s ability to discriminate between high and low Ki-67 expression GBM patients, complementing the quantitative performance metrics.

**FIGURE 3:**
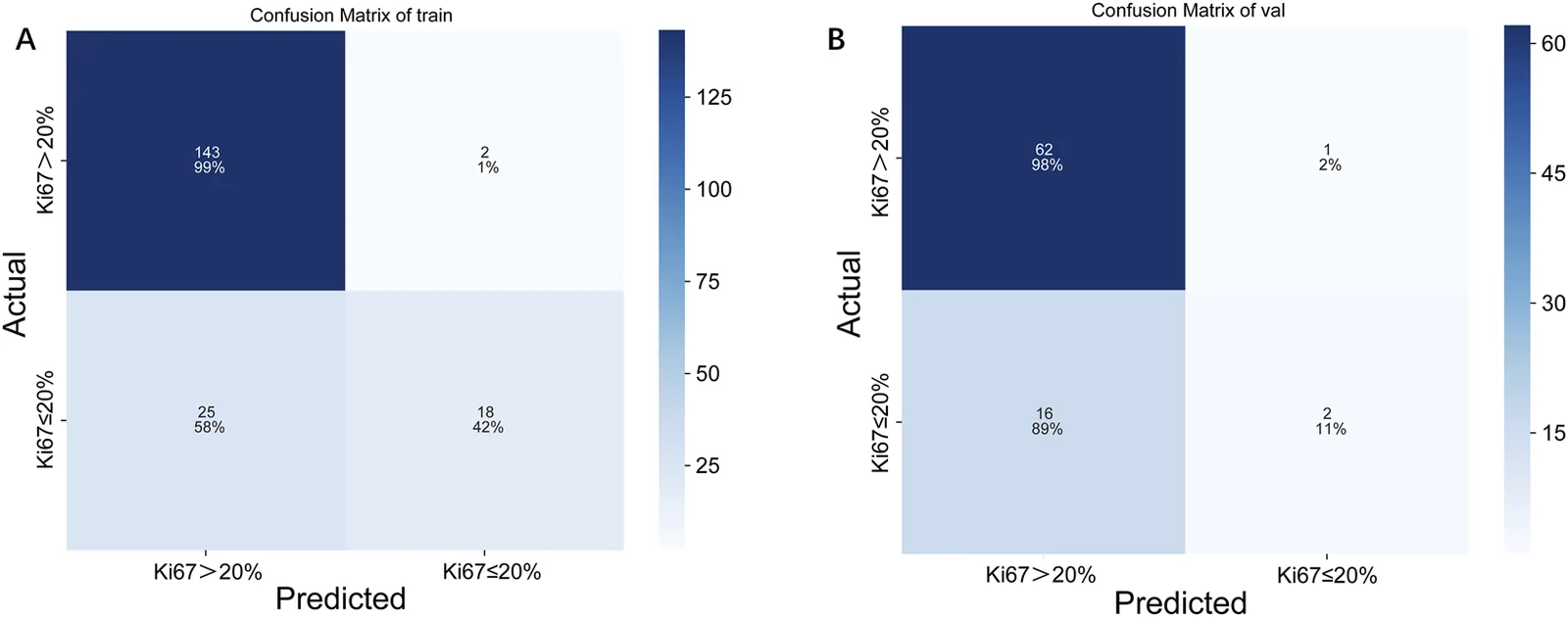
**(A)** Confusion matrix of testing cohort, **(B)** Confusion matrix of validation cohort.

### 3.5 Explainability of the combined model

SHAP analysis identified the core features driving model predictions. The top five key features ranked by contribution were as follows: LBP2D gray level co-occurrence matrix autocorrelation of T2 (lbp2D_glcm_Autocorrelation_T2), original gray level co-occurrence matrix information measure of correlation 2 of T1 (original_glcm_Imc2_T1), wavelet LLH first-order kurtosis of FLAIR (waveletLLH_firstorder_Kurtosis_FLAIR), LBP3Dk first-order skewness of T1 (lbp3Dk_firstorder_Skewness_T1), and LBP3Dm2 gray level zone matrix small area emphasis of FLAIR (lbp3Dm2_glszm_SmallAreaEmphasis_FLAIR), Figure 4.

**FIGURE 4:**
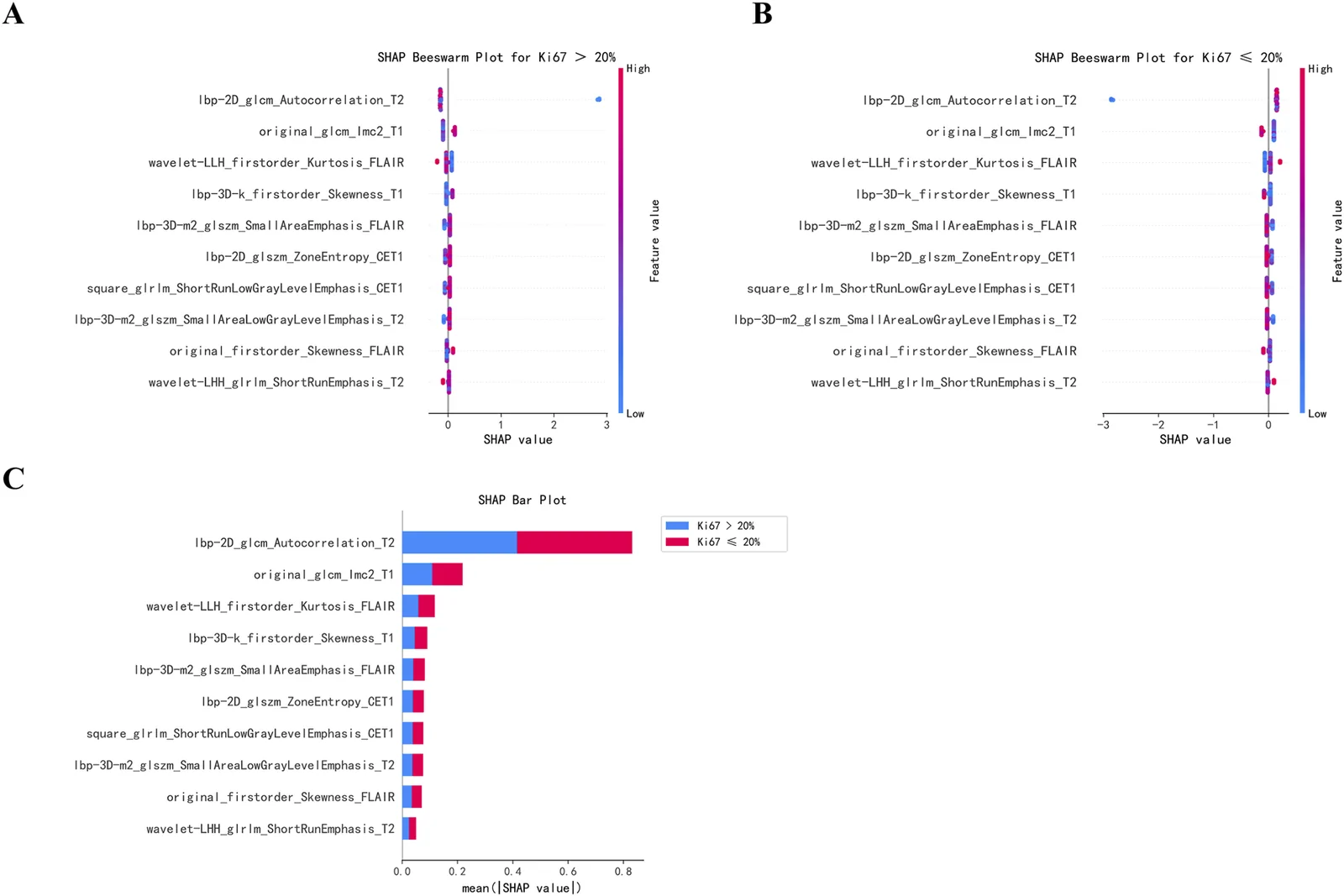
**(A)** SHAP for Ki-67 > 20%; **(B)** Ki-67 ≤ 20%; **(C)** SHAP for combined.

Autocorrelation reflects spatial similarity of gray levels in tumor regions, which is closely related to dense cell arrangement and intratumoral heterogeneity. Information measure of correlation 2 captures textural complexity and heterogeneity, indicating the activity of cell proliferation. Kurtosis describes the steepness of signal distribution, reflecting microstructural alterations such as necrosis and hemorrhage. Skewness characterizes asymmetry of signal distribution, indicating deviation from normality within the tumor. Small area emphasis quantifies the proportion and distribution of tiny hyper- or hypo-intense foci, indirectly reflecting microstructural fragmentation and invasiveness. These features contributed stably to the differentiation between high Ki-67 expression (>20%) and low Ki-67 expression (≤20%), providing interpretable biological and imaging evidence for the model.

## 4 Discussion

In this study, we developed and internally validated a multi-sequence MRI-based radiomics machine learning model to non-invasively estimate Ki-67 expression levels in glioblastoma patients. Our results demonstrate that the combined model, integrating radiomic features from T1WI, T2WI, FLAIR, and CET1WI sequences, achieved superior predictive performance compared with single-sequence models, with an AUC of 0.915 in the training set and 0.746 in the test set (Li et al., 2024; Dong et al., 2025). This finding underscores the complementary nature of multi-parametric MRI data, as different sequences capture distinct aspects of tumor biology, including cellularity, edema, necrosis, and vascularity, which collectively contribute to a more comprehensive characterization of the proliferative microenvironment (Ren et al., 2025). However, the decline in performance from the training to test set indicates that the model should be considered exploratory until validated in independent cohorts. The high sensitivity (0.930) and negative predictive value (0.972) in the training set suggest potential usefulness for Ki-67 risk stratification, but do not establish that radiomics-predicted Ki-67 can guide aggressive therapeutic regimens. Furthermore, SHAP analysis revealed that key features such as LBP2D_glcm_Autocorrelation from T2 and waveletLLH_firstorder_Kurtosis from FLAIR were among the top contributors, highlighting the biological interpretability of the model by linking textural heterogeneity and signal distribution to underlying cellular proliferation and tissue microarchitecture (Chen et al., 2024).

Glioblastoma (GBM) is the most common and aggressive primary malignant brain tumor in adults, characterized by a dismal prognosis despite multimodal treatment regimens including maximal safe resection, radiotherapy, and chemotherapy (Tanaka et al., 2013; Pan et al., 2025). The proliferative activity of GBM, a key determinant of its aggressive behavior, is commonly assessed via the Ki-67 labeling index, which serves as a robust biomarker for tumor proliferation and prognosis (Shirahata et al., 2018). However, the preoperative, non-invasive determination of Ki-67 expression levels remains a significant clinical challenge. While previous studies have employed radiomics based on single-sequence MRI to predict Ki-67 expression in gliomas, these approaches often suffer from limited predictive performance due to the inability to capture the complex, heterogeneous information embedded across multiple imaging sequences (Li et al., 2024). Our study introduces a novel multi-sequence MRI-based radiomics machine learning model that integrates features from T1WI, T2WI, FLAIR, and CET1WI. This integrated approach addresses a critical knowledge gap by systematically demonstrating that a combined model, leveraging the complementary information from all four sequences, significantly outperforms any single-sequence model in predicting Ki-67 expression levels in GBM. Compared to prior work that often focused on single or two-sequence analyses (Ohgaki and Kleihues, 2013; Prados et al., 2015; Lopes Abath Neto and Aldape, 2021), our findings provide the first comprehensive evidence that the synergistic combination of multi-parametric MRI data yields a substantial improvement in discriminative power, with the Combined model achieving an area under the curve (AUC) of 0.915 in the training set and 0.746 in the test set. This performance is notably superior to that of individual sequence models, particularly the CET1 sequence (AUC of 0.499 in the test set), which alone showed poor predictive capability, underscoring the necessity of a multi-sequence approach for capturing the full spectrum of tumor biology.

The clinical implications of our findings should be interpreted cautiously. Ki-67 reflects proliferative activity and may contribute to preoperative biological risk assessment, but it is not currently used as a stand-alone marker for GBM treatment selection. In addition, the present study did not include analyses linking measured or radiomics-predicted Ki-67 to overall survival, progression-free survival, recurrence, or treatment response. Therefore, our model should be regarded as an adjunctive radiomics tool for estimating Ki-67 status rather than evidence for modifying temozolomide scheduling, radiotherapy strategy, or clinical trial allocation. Decision curve analysis suggested possible net benefit across selected thresholds, but prospective outcome-based validation is required before integration into routine clinical workflows.

The interpretability of our model, facilitated by SHapley Additive exPlanations (SHAP) analysis, provides transparent, biologically plausible insights into the imaging features driving predictions. The identification of key features such as LBP2D_glcm_Autocorrelation from T2 and original_glcm_Imc2 from T1 highlights the importance of textural heterogeneity and spatial gray-level similarity, which may reflect dense cellularity and intratumoral heterogeneity characteristic of high Ki-67 expression20. Similarly, the contribution of wavelet-derived features such as kurtosis and skewness from FLAIR and T1 sequences may capture microstructural alterations, including necrosis, hemorrhage, and irregular cellular architecture11. This level of explainability may help radiologists and neuro-oncologists evaluate model behavior and supports its use as a complementary research tool. Moreover, the model’s reliance on multi-sequence MRI data, which is routinely acquired in standard GBM imaging protocols, supports practical feasibility without requiring additional specialized imaging sequences.

## 5 Limitations

Despite the promising performance of our multi-sequence radiomics model, several limitations warrant acknowledgment. First, this was a retrospective single-center study with a relatively modest sample size, which may introduce selection bias and limit the generalizability of our findings to broader populations. Although we employed internal validation *via* a 7:3 split, the observed drop in AUC from the training (0.915) to the test set (0.746) suggests potential overfitting or cohort-specific characteristics; therefore, model robustness should be interpreted cautiously and requires larger, multi-center prospective validation. Second, Ki-67 was analyzed using a single patient-level pathological label derived from routine clinical pathology reports. Because GBM is highly spatially heterogeneous, Ki-67 expression may differ across enhancing tumor, non-enhancing tumor, necrotic or perinecrotic regions, and infiltrative margins. This creates a potential mismatch between whole-tumor 3D radiomic features and the sampled pathological ground truth. Third, the 20% cutoff was selected according to published Ki-67 stratification literature and local reporting practice, but Ki-67 is biologically continuous; alternative thresholds and continuous-variable modeling should be evaluated in future studies when harmonized raw Ki-67 and radiomic feature data are available. Fourth, this study did not include overall survival, progression-free survival, recurrence, or treatment response data; consequently, the clinical utility of measured or radiomics-predicted Ki-67 for treatment decision-making could not be directly assessed. Fifth, while manual segmentation by experienced radiologists ensured accuracy, it remains time-consuming and susceptible to inter-observer variability; future studies should explore automated segmentation algorithms to enhance reproducibility. Finally, although SHAP analysis provided biological interpretability linking texture features to tumor heterogeneity, the underlying molecular mechanisms connecting specific radiomic signatures to Ki-67 proliferation indices remain hypothetical and require further histopathological correlation.

## 6 Conclusion

In conclusion, this study demonstrates that a machine learning model integrating radiomic features from multi-sequence MRI can non-invasively estimate Ki-67 expression levels in glioblastoma patients. The combined model outperformed single-sequence approaches and achieved favorable discrimination, suggesting potential value for preoperative biological risk stratification. By identifying imaging features related to intratumoral heterogeneity and microstructural fragmentation, our work supports the feasibility of linking radiological phenotypes with molecular proliferation status. These findings suggest that multi-sequence radiomics may serve as a complementary, non-invasive adjunct to pathological assessment. Future research should focus on external validation, continuous or alternative-threshold Ki-67 modeling, spatially matched radiology-pathology correlation, and outcome-based assessment before clinical decision-support implementation.

## Data Availability

The original contributions presented in the study are included in the article/supplementary material, further inquiries can be directed to the corresponding authors.
